# Early-Life Experiences and Caregiver Status in Mid- and Later Adulthood

**DOI:** 10.1093/geroni/igaf122.922

**Published:** 2025-12-31

**Authors:** Florence Johnson, Maria Roche-Dean, Yiqing Qian, Jacobs Moyosoreoluwa, Richard Gonzalez, Sheria Robinson-Lane

**Affiliations:** University of Michigan School of Nursing, Ann Arbor, Michigan, United States; Kirkhof College of Nursing, Grand rapids, Michigan, United States; Johns Hopkins University School of Nursing, Baltimore, Maryland, United States; Wayne State University Department of Psychology, Detroit, Michigan, United States; University of Michigan, Ann Arbor, Michigan, United States; University of Michigan School of Nursing, Ann Arbor, Michigan, United States

## Abstract

The selection of caregivers within families is a complex process influenced by various early-life factors, such as parental relationships, a history of physical abuse, and care provided by grandparents. Understanding how these contextual factors lead to the role of the adult family caregiver is crucial for developing effective support systems for caregivers. Using data from the 2019 Health and Retirement Study Life History survey, this study described early family life experiences below age 18 among caregivers and non-caregivers. Among the 2,849 respondents, we found 23% (n = 650) reported ever providing unpaid care to an adult family or friend for at least 6 months. Female respondents (29%) were more likely to be caregivers than male respondents (15%). We found respondents who lived with grandparents before 18 (27%) were more likely to be caregivers. Notably, about 32% of respondents who reported physical abuse before age 18 identified as caregivers, higher than the percentage of caregivers among those who did not report abuse (24%). Furthermore, we found those with good relationships with mothers were less likely to be caregivers (23% vs 27%). No difference in relationships with fathers was found (24%). Overall, these results highlight caregivers’ diverse backgrounds and experiences, underscoring the need for targeted support and resources to address the unique challenges different caregiving populations face. Further research is needed to understand the underlying factors driving these caregiving patterns and to develop interventions that can alleviate the burden on caregivers, particularly those with histories of trauma or living in multigenerational households.

